# Highlighting the Compositional Changes of the Sm_2_O_3_/MgO-Containing Cellulose Acetate Films for Wound Dressings

**DOI:** 10.3390/polym14224964

**Published:** 2022-11-16

**Authors:** Yousef A. A. Alghuwainem, Mohamed Gouda, Mai M. Khalaf, Fakiha El-Taib Heakal, Hanan A. Albalwi, Abraham Elmushyakhi, Hany M. Abd El-Lateef

**Affiliations:** 1 Department of Veterinary Public Health and Care, College of Veterinary Medicine, King Faisal University, Al-Ahsa 31982, Saudi Arabia; 2 Department of Chemistry, College of Science, King Faisal University, Al-Ahsa 31982, Saudi Arabia; 3 Chemistry Department, Faculty of Science, Sohag University, Sohag 82524, Egypt; 4 Chemistry Department, Faculty of Science, Cairo University, Giza 12613, Egypt; 5 Department of Chemistry, College of Science and Humanities in Al-Kharj, Prince Sattam Bin Abdulaziz University, Al-kharj 11942, Saudi Arabia; 6 Department of Mechanical Engineering, College of Engineering, Northern Border University, Arar 73213, Saudi Arabia

**Keywords:** wound dressing, cellulose acetate, wound bandages, Sm_2_O_3_, MgO

## Abstract

The development of wound dressing materials with appropriate specifications is still a challenge to overcome the current limitations of conventional medical bandages. In this regard, simple and fast methods are highly recommended, such as film casting. In addition, deliverable nanoparticles that can act to accelerate wound integration, such as samarium oxide (Sm_2_O_3_) and magnesium oxide (MgO), might represent a potential design with a novel compositional combination. In the present research, the casted film of cellulose acetate (CA) was mixed with different ratios of metal oxides, such as samarium oxide (Sm_2_O_3_) and magnesium oxide (MgO). The tests used for the film examination were X-ray diffraction (XRD), Fourier-transform infrared spectroscopy (FTIR), and scanning electron microscopy (SEM). The SEM graphs of CA films represent the surface morphology of Sm_2_O_3_@CA, MgO@CA, and Sm_2_O_3_/MgO/GO@CA. It was found that the scaffolds’ surface contained a high porosity ratio with diameters of 1.5–5 µm. On the other hand, the measurement of contact angle exhibits a variable trend starting from 27° to 29° for pristine CA and Sm_2_O_3_/MgO/GO@CA. The cell viability test exhibits a noticeable increase in cell growth with a decrease in the concentration. In addition, the IC_50_ was determined at 6 mg/mL, while the concentration of scaffolds of 20 mg/mL caused cellular growth to be around 106%.

## 1. Introduction

Upon the environmental factors, wounds and injuries have become easy to occur in the body shield (skin) [1]. Skin is the first destination for protection against accidents [1]. Healing skin wounds in a short period is still a challenge for researchers [2]. The wounds were traditionally treated with antibiotics to prevent the wounds from being infected [3]. On the other hand, excessive use of antibiotics for a long time affects the healthcare system [4].

Wound healing is a major complex process that includes four stages: hemostasis, inflammatory reactions, cell proliferation, and tissue remodeling [5]. The traditional bandages could be enhanced with biomedical materials to cover all needs of the high-impact therapy [2,6]. Film casting is one of the ways of manufacturing bandages that may stimulate live cells. Wound dressings may be fabricated with thin polymeric films, which are suitable for wounds because of their biocompatibility, good adhesion, and flexibility [7].

Cellulose acetate (CA) is a chemically and thermally stable polymer [8]. It exhibits good biocompatibility and adequate degradation behavior [8]. CA is a non-toxic, renewable polymer having low chemical reactivity, and is suitable for medical applications, such as wound dressings [9,10,11]. Although CA has excellent hydrophilicity and high capability, pure CA has low bioactivity, which affects skin repair [10,12].

In general, lanthanide metal oxides have special optical properties, and they are used in electronics, laser, and photocatalysts applications. Samarium oxide (Sm_2_O_3_) is an important rare earth metal oxide [13]. It has attractive characteristics, such as pH sensing and good gas detection [13]. Nanoparticles of Sm_2_O_3_ are also utilized in solar cells, infrared absorption, catalyst, and biological sensors. In addition, they are thermally stable (melting point 2335 °C) [14]. Sm_2_O_3_ nanoparticles can be prepared by several methods, but one of the methods is thermal decomposition. The ability to be prepared by thermal decomposition with desired properties, such as spherical nanoparticles with an average size of around 50 nm, is important due to the low cost and large production of thermal decomposition [15]. It is used in various biomedical applications, such as an antibacterial agent [16] and also as a bone-substituting material [17]. Balamurugan et al. [18] have synthesized Sm-doped CeO_2_ nanoparticles by the hydrothermal process. They reported antibacterial activity against different bacterial species, such as *E. coli*, *B. cereus*, *S. aureus*, and *S. Typhi*, with inhibition zones reaching 22, 20, 25, and 24 mm, respectively. That indicates its ability to prevent inflammation of wounds besides being non-toxic when loaded in the biocompatible CA. Magnesium oxide (MgO) is one of the anti-oxidant materials [19]. It has wide use in biological applications due to its nontoxicity and excellent adsorption capacity [19,20]. It has suitable properties for cellular protection and vital protection against radical damage [21].

Graphene oxide (GO) is one of the common materials used in biomedical applications such as tissue engineering and drug delivery [22,23]. It has several essential properties, such as high mechanical strength, hydrophilicity, biocompatibility, and good anti-microbial behavior [22,24]. Furthermore, the incorporation of GO nanosheets through the scaffolds might act as a reinforcement agent to support their mechanical resistance against the applied stresses. In addition, the hydrophilic behavior of GO due to the connected oxyanion groups might provide significant additional modification through the surface of CA [18]. This effect is important to activate the adhesion tendency of the formed scaffolds with the cellular environments.

In the study by Kalaycıoğlua et al. [25], CA was fabricated with chitosan (Chit) and cerium oxide (CeO_2_) in the form of films. For all those films, the thermal gravimetric analysis (TGA) showed four stages starting with ~80 °C, then followed by ~180 °C and ~220 °C, and finally, the degradation stages ended at ~350 °C. As concluded, the addition of CeO_2_ to CA and Chit represents high thermal stability and low weight loss. The SEM images appeared with a porous surface [25].

Moreover, using the electrospinning technique Ullah et al. [3] have loaded Blumea balsamifera oil (BB oil) with CA in the form of nanofiber. The surface morphology represented fibers with a diameter of ~324 ± 94 nm. The addition of BB oil showed a noticeable increase in the fibers’ diameter to 362 ± 162 nm for CA/BO10, 420 ± 178 nm for CA/BO15, and 448 ± 180 nm for CA/BO20. This increase in the fibers’ diameters has led to a decrease in their porosity.

It is worth mentioning that pure CA has a lot of advantages, such as high biocompatibility and stability. Nevertheless, it lacks appropriate bioactivity in cellular environments. This bioactivity is dependable on surface features, which could tailor via the compositional variation. This means that particles addition such as Sm_2_O_3_ and MgO might promote the topography of CA to be rougher than the pure phase of CA. These changes in surface topology are essential for controlling the hydrophilic behavior of the scaffold. Additionally, the Mg ions are essential components of the human body, as it plays an important role in manipulating mineral metabolism. Furthermore, it supports the mechanical properties of hard tissues. In the meantime, Sm_2_O_3_ is a good candidate for antibacterial effects, which might be important for the new implant materials. Besides, Sm_2_O_3_ can improve the sensing properties of the designed scaffolds, which is highly important for smart bandages. Consequently, gathering those metallic oxide components, such as Sm_2_O_3_ and MgO, to be incorporated into CA scaffolds might represent a proper multifunctional design with topographical features and compositional components to be a good candidate for accelerating injury integration. The main purpose of this work is to investigate the performance of CA wound scaffolds loaded with Sm_2_O_3_ and MgO. Although CA is a biocompatible and hydrophilic material, it has low mechanical strength and poor bioactivity. These defects could be improved by the addition of GO and MgO according to their wide biological characteristics. Therefore, the current study aims to scrutinize the optimum physicochemical properties of the CA scaffolds encapsulated with Sm_2_O_3_ and MgO nanoparticles. The physicochemical properties will be studied, including the structure, morphology, and contact angle, besides the swelling behavior and the biological responses of the cells towards the scaffolds in vitro.

## 2. Materials and Methods

### 2.1. Materials

The used basic materials are samarium oxide (99%) (50–80 nm), magnesium oxide (99%) (30–70 nm), graphite (98%), hydrochloric acid (HCl, 36.5%), potassium permanganate (KMnO_4_, 98%), sulphuric acid (H_2_SO_4_, 98%), H_3_PO_4_, H_2_O_2_, cellulose acetate (M = 45,000 g/mol), acetone. These materials were obtained from Alpha India.

### 2.2. Thin Film Fabrication

The nanoparticles like Sm_2_O_3_ and MgO were used as supplied. Graphene oxide (GO) was prepared in the lab using the modified Hummer’s method. A 3 g graphite powder was added to a 9:1 mixture of concentrated H_2_SO_4_/H_3_PO_4_ with continuous stirring for 5 min. Then, 18 g of KMnO4 was added to the previous mixture with continuous stirring for 12 h. Then, 3 mL of H_2_O_2_ was added and mixed for 1 h. The solution was washed with 30% HCl, then with distilled water, and with ethanol, and dried in the furnace—a solution of 7 wt.% CA was prepared. Five stock samples of CA were planned, each one containing 20 mL. The first one was kept pure as CA without any additives. The metal oxide nanoparticles were added to the other four CA solutions with a total weight of 0.25 g for each film. It could be noticed that the additives were dropped into each bottle, and the solutions in each bottle were stirred using a magnetic stirrer for 1 h to get well-dispersed solutions [2,9]. In addition, the contributions of CA and metal oxide nanoparticles are (1) pure CA, (2) 0.25 g of Sm_2_O_3_ added to CA, (3) 0.25 g of MgO added to CA, (4) 0.125 g of Sm_2_O_3_ and 0.125 g of MgO were added to CA, (5) 0.1 g of Sm_2_O_3_, 0.1 g of MgO and 0.05 g of GO were added to CA. Then, the samples were cast into a petri dish and left in the drier furnace till complete dryness.

### 2.3. Characterizations

#### 2.3.1. XRD Measurements

X-ray diffraction (XRD) has been used to identify the formed phases. The specifications of the XRD apparatus were analytical-x’ Pertpro with Cuk_α1_, the Netherlands. All XRD curves were scanned in 5° ≤ 2θ ≤ 70° with a step size of 0.02° and a step time of 0.5 s.

#### 2.3.2. FT-IR Measurements

Fourier transforms infrared (FTIR) spectra were used for all samples by (Perkin-Elmer (FTIR). The analysis was scanned in the range of 400–4000 cm^−1^ via a transmittance mode. The films were scanned without additional KBr for dilution.

#### 2.3.3. Morphology Investigation

The surface morphology was examined through a scanning electron microscope (SEM) (QUANTA-FEG250, Netherlands). The operating voltage was around 10 kV. Moreover, Energy dispersive X-ray (EDX) was performed using the same SEM instrument.

#### 2.3.4. Contact Angle Test

The contact angle was evaluated by a custom system via water drops. A sample of 1.0 cm^2^ of each cast film was fixed versus the camera. Moreover, the images were taken when the drop of water was released.

#### 2.3.5. Swelling Degree Study

The swallow ability of the scaffolds has been done via soaking a constant weight of each sample (0.02 g) through 50 mL of deionized water for 12 h at 37 °C. During this time, the sample was taken out every period to be weighted. The total weights were compared with the dried weights to get the swelling degree for each scaffold.

#### 2.3.6. In Vitro Cell Viability Tests

The cell viability was tested with normal lung cells (WI-38). The cell lines information is as follows: Database Name: ATCC, Accession Numbers: WI-38 (ATCC CCL-75). The WI-38 cell lines were isolated from the lung tissue of a 3-month-old female embryo, Organism: Homo sapiens, human, Cell Type: Fibroblast, Tissue: Lung, Age: 3 months’ gestation, Gender: Female, Morphology: Fibroblast, Growth properties: Adherent, Disease: Normal.

The culture was in Gibco medium. The samples were weighed individually and then soaked in sterilized water for 24 h. The solution was serialized in 96 well-plate starting from the higher concentration to the lowest one of the scaffold. The scaffold was immersed in a tube containing sterilized water was a concentration of 4000 µg/mL. Then, the plates were incubated for 72 h at 37 °C. after that, the media was removed, and the (3-(4,5-dimethylthiazol-2-yl)-2,5-diphenyltetrazolium bromide) (MTT) was added to measure the cell viability using the optical density.

## 3. Results and Discussion

### 3.1. XRD

**Figure 1:**
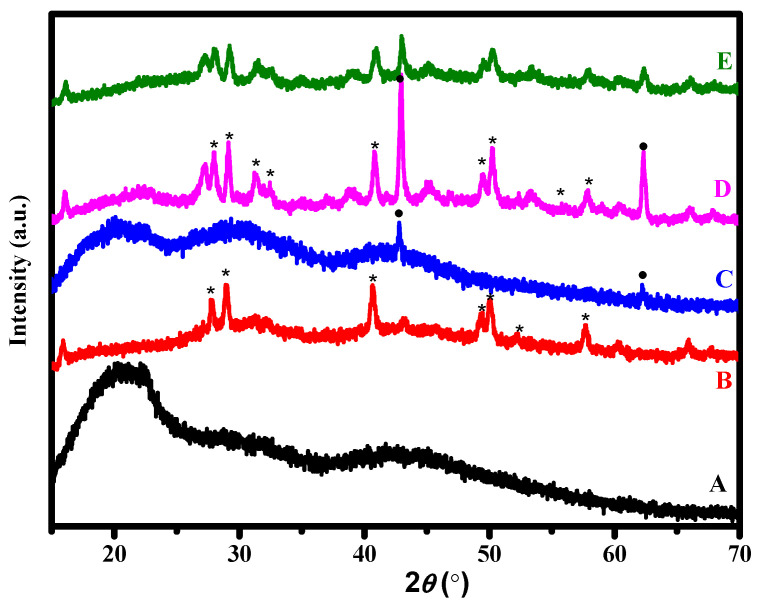
XRD patterns of different ratios of nanoparticles added to CA; (*: Sm_2_O_3_ and ●: MgO); (A) CA, (B) Sm_2_O_3_@CA, (C) MgO@CA, (D) Sm_2_O_3_/MgO@CA, and (E) Sm_2_O_3_/MgO/GO@CA.

X-ray diffraction is used to determine the crystallinity and the compositions of the materials. As obvious in Figure 1, the XRD graph shows that CA exhibits several broad halos or hump peaks, which may refer to its low crystalline phase [26]. The reason behind these halos is the non-long-range order. Therefore, there are no well-defined scattering planes leading to the broadness of the peaks. However, the pattern indicates a hump peak at around 2-theta = 20°. The Sm_2_O_3_ display sharp peaks at 27.7°, 28.9°, 40.7°, 49.3°, 50.05°, and 57.6°. The miller indices of these peaks show that the Sm_2_O_3_ was crystallized in a cubic structure (a = b = c) [13]. The diffraction peaks of MgO were maintained at 42.7° and 62.2° associated with diffraction planes of (002) and (022), respectively [19]. Likewise, the structure of MgO tends to be cubic [19]. Moreover, it can be noticed that the crystallinity of MgO is relatively low compared to Sm_2_O_3_. Therefore, the humps of the amorphous phase of CA are noticeable and clear. On the other hand, the low contribution of GO might limit its significant effects on the other structures. The sharp peaks performed at the curve of pure CA and the combination of CA with MgO. The curve of Sm_2_O_3_ represents sharp peaks that define the major crystallinity. That means CA shows an amorphous phase, MgO has relatively low crystallinity, and Sm_2_O_3_ shows a higher crystallinity, while GO cannot be detected due to its low quantity. The existence of low crystalline nanoparticles and amorphous CA can directly affect the roughness of the surface. Therefore, enhancing the attachment of cells leads to low cytotoxicity.

### 3.2. FTIR

**Figure 2:**
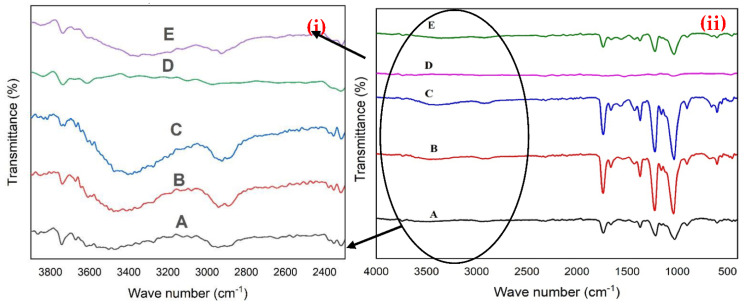
FTIR of CA with different additives of nanoparticles; (A) CA, (B) Sm_2_O_3_@CA, (C) MgO@CA, (D) Sm_2_O_3_/MgO@CA, and (E) Sm_2_O_3_/MgO/GO@CA. (**i**) FTIR spectra of the range 2400–4000 cm^−1^, while (**ii**) represents the whole spectra from 400–4000 cm^−1^.

The FT-IR spectra were examined for all compositions in the range of 400–4000 cm^−1^ (Figure 2i,ii, Table 1). In the FTIR spectra for CA samples, the band at 906 cm^−1^ represents C–OH stretching [27]. The bands observed at 1025, 1159, and 1212 cm^−1^ corresponded to the C–O functional group in the absorption region, –CH groups of CA, an alkoxyl stretch of the ester (C–O–C) [8,28]. In addition, the bands at 1369, 1426, 1662, and 1742 cm^−1^ referred to methyl bending (C–CH_3_), –CH groups of CA, C=O stretch, and stretching vibrations of carbonyl functional groups [8,27,28,29]. In addition, the bands of –CH_2_ stretching and hydroxyl stretching vibration were exhibited at 2943 and 3742 cm^−1^ [30,31]. On the other hand, the FTIR spectra for Sm_2_O_3_ have a band at 3454 cm^−1^, which indicates the stretching vibration of the (–OH)group [13]. The determined bands for MgO represented at 476, 558, and 658 cm^−1^, which corresponded to Mg–O–Mg bonds indicated by the stretching vibration mode and MgO vibrations [19,32]. The band at 3439 referred to the hydroxyl groups (–OH) [33]. The spectrum of Sm_2_O_3_/MgO@CA exhibits bands at 1054, 2972, 3393, and 3836 cm^−1^, which referred to C–O, C–H stretching, (–OH) group, and O–H bonds [8,33,34,35]. The addition of GO represents two bands at 1558 and 2926 cm^−1^, which are assigned to C=C bonds and C–H stretching [34,36]. The bands around 1000–1100 cm^−1^ seem to be relatively very high compared with the OH bands. Therefore, the OH bands might be covered, or their peaks look with low relative intensity. Here, the region of OH bands is magnified to be clearer, as obvious in Figure 2i.

### 3.3. Surface Morphology

**Figure 3:**
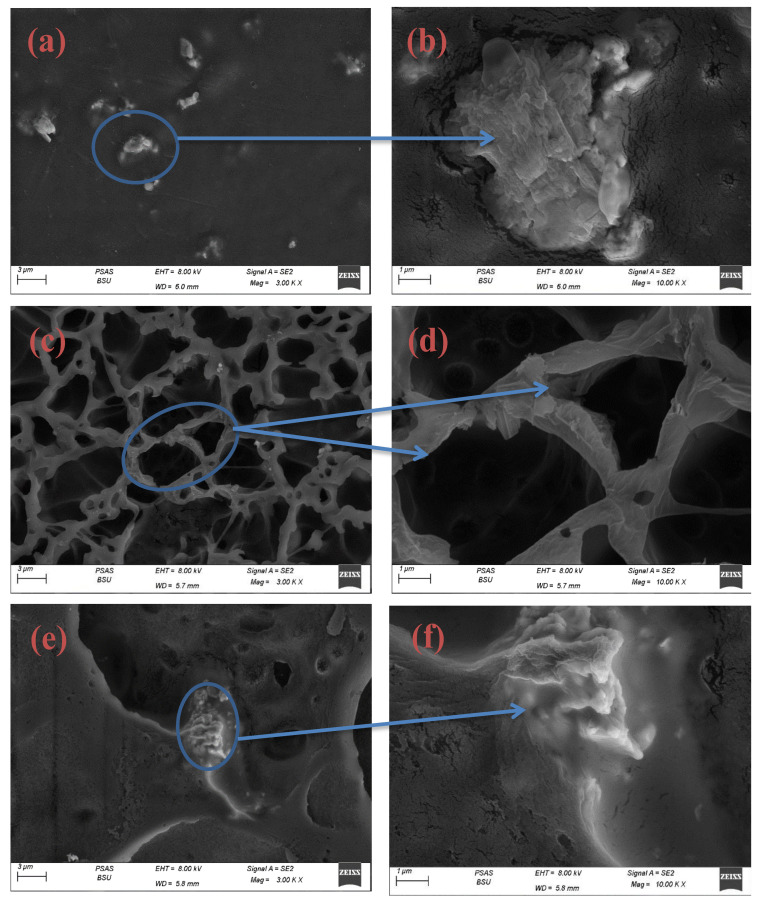
SEM micrograms of CA film casting: (**a**,**b**) Sm_2_O_3_@CA (**c**,**d**) Sm_2_O_3_/MgO@CA, (**e**,**f**) Sm_2_O_3_/MgO/GO@CA.

The term (SEM) refers to a scanning electron microscope that is usually used to examine the surface morphology of the films. The topography of Sm_2_O_3_@CA film is shown in Figure 3a,b. It illustrates that the surface tends to be smooth. However, some blocks containing pores and cracks tend to be slightly rough. As obvious in Figure 3c,d of Sm_2_O_3_/MgO@CA film, it is shown that with the addition of MgO, the surface became filled with pores, which have diameters in the range of 1.5–5 µm and lengths in the range of 0.5–9 µm. The borders of pores are illustrated with rough textures, which might represent strong adhesion [37]. Figure 3e,f shows the effect of adding GO exhibits a porosity ratio less than the previous one. The diameters of pores are between 0.5 and 5 µm. In addition, the surface roughness seems to be decreased according to the 2d surface of the GO nanosheet with low crystallographic defects, which might regulate the chemical interactions with good conductivity. Sivasankari et al. [38] prepared a membrane of CA loaded with hydroxyapatite (HAP). They found high porosity in the membrane of CA, which is important in biomedical applications. David et al. [39] prepared CA-collagen containing multi-wall carbon nanotubes (MWCNTs) decorated by Titanium dioxide (TiO_2_). The porous nature of CA film is also detected by SEM microscopy. Moreover, Liakos et al. [40] prepared CA-essential oil nanocapsules for antimicrobial biomedical applications. They reported that SEM micrographs captured the porosity in the CA film.

### 3.4. EDX Analysis

**Figure 4:**
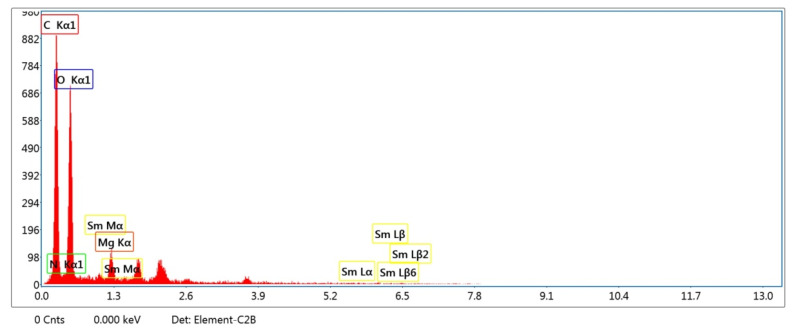
EDX analysis of the scaffold containing Sm_2_O_3_/MgO/GO@CA.

Energy Dispersive X-ray (EDX) is an elemental test to determine the ratios of the contributed elements. According to Table 2 and Figure 4 the major ratios referred to oxygen and carbon by 48.84 and 43.17%, which were assigned to the high contribution of CA, besides the presence of GO. In addition, magnesium is determined by a ratio of about 2.74%. On the other hand, the minor concentration referred to Sm by 1.66%. The existence of the elements was approved by SEM test, and the nanoparticles were successfully fabricated on the surface of the films.

### 3.5. Contact Angle

**Figure 5:**
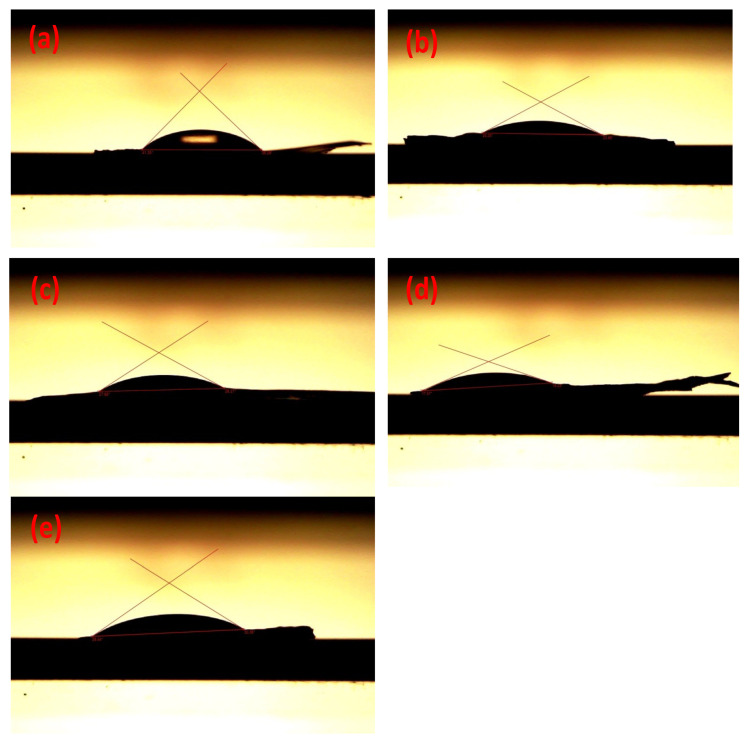
The contact angle of CA with different additives of metal oxides: (**a**) CA, (**b**) Sm_2_O_3_@CA, (**c**) MgO@CA, (**d**) Sm_2_O_3_/MgO@CA, (**e**) Sm_2_O_3_/MgO/GO@CA.

The contact angle is an essential examination test to determine the ability of the casted film to interact with drops of distilled water on the film surface. As obvious in Figure 5a, the pure CA exhibits an average contact angle of 39.81°. The addition of Sm_2_O_3_ leads to reducing the angle to 24.8°, as in Figure 5b. On the other hand, the addition of MgO causes a slight increase to be at 24.9°. However, as shown in Figure 5d, the mixture of different contributions of the nanoparticles with CA exhibit a noticeable decrease till 23.9°. Then, the addition of GO to these composites led to a significant increase, and the contact angle was recorded at 29.4°.

The addition of Sm_2_O_3_ might promote the formation of crystal defects on the surface of CA. the effect of MgO on the surface of CA is approximated to the effect of Sm_2_O_3_. The combination of Sm_2_O_3_ and MgO might induce higher defects than the addition of each one in a single phase due to the difference in crystallographic orientation. The additive of GO nanosheet might recover the addition of the other nanoparticles because of its low defects compared with Sm_2_O_3_ and MgO. As a result, the degradation behavior might be promoted with the reduction of hydrophobicity that helps cells to attach, divide and promote wound healing.

In a recent study by Elsherbiny et al. [41], the contact angle for pure CA was recorded at 81°, which may pretend its hydrophobic nature. The composite of CA/lignin showed a noticeable decrease in the angle and was determined at 73°. A variety of lignin concentrations represents contact angles at 45°, 30°, and 8° for L29, L37, and L44. This significant decrease may be assigned to the hydrophilic nature of the lignin structure and the accelerated biodegradability of the scaffolds. Guezguez et al. [42] have prepared CA films, and they reported that the contact angle with water was around 54° before the modifications. Mahdavi et al. prepared CA with different concentrations of different blends. They reported that the CA contact angle could be in the range of 25°–70.4°. Tsekova et al. [43] have reported the contact angle of CA fibrous materials at 123.10 ± 2.0°, which regulates the hydrophobic nature of this material. At the same time, the contact angles of the compositions CA/PVP and Curc/CA + PVP were evaluated at 36 ± 3.5° and 14.8 ± 1.7°, respectively, due to the hydrophilization characteristics of PVP. As a result, the hydrophilic behavior of PVP led to a reduction of the surface tension, which enhances the contact angle of CA. The literature refers that the contact angle can be affected by the quantity of CA and its solvent, in addition to the preparation method and the incorporated particles.

### 3.6. Swelling Degree

**Figure 6:**
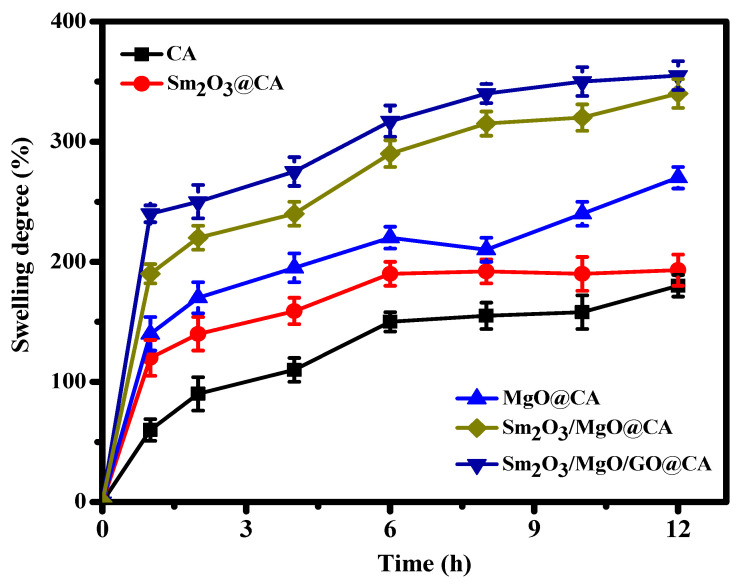
Swallowability degree of CA cast films containing different dosages of Sm_2_O_3_ and MgO nanoparticles.

The ability of the scaffold to interact with the injury solutions is essential to keep the moisture of the wound and to avoid dehydration. As well, inhibition of inflammation might be achieved by controlling the solution path through the scaffolds that cover the wound area. In this regard, the swelling degree of the scaffolds has been carried out, as illustrated in Figure 6. As may be seen, the ability to adsorb water increased with the additional nanoparticles of Sm_2_O_3_ and MgO. The pure CA started its plateau after around 6 h of soaking with a degree of 150 ± 8%. Furthermore, the highest swelling degree was obtained with the scaffold containing Sm_2_O_3_/MgO/GO@CA with a degree of 340 ± 12% after 12 h of socking. The boosting in the swelling degree with the nanoparticle’s modification might reflect the ability of these additives to manipulate the topographical features of the scaffold. Hence, controlling the additional nanoparticles through the scaffolds leads to a high adjustment of the surface roughness and, thus, good controlling of the wettability and swallowability. The biodegradation of these scaffolds through the biological environment also might be controlled via surface modification. Consequently, it is noticed that structural and morphological features are good tools to control the scaffold’s response toward the biological milieu.

### 3.7. Cell Viability

**Figure 7:**
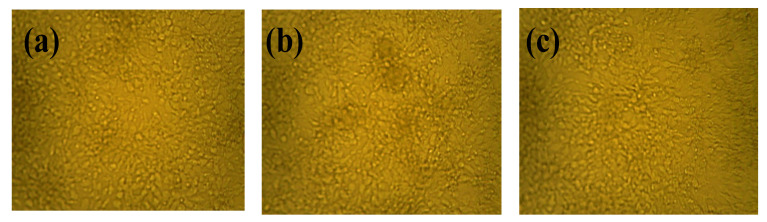
The images of viable cells with the optical microscope of Sm_2_O_3_/MgO/GO@CA (**a**–**c**); (**−−−−−**500 µm).

**Figure 8:**
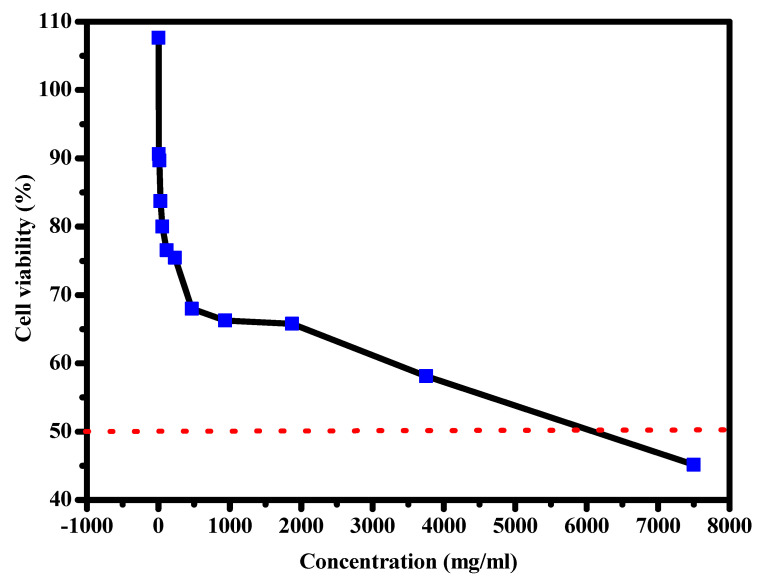
Cell viability graph of CA cast film with different contributions of nanoparticles after culture with normal lung cells (D).

The cell viability test is used to examine the viability of cells and indicates the biocompatibility of scaffolds within normal cells. As obvious in Figure 7a–c, the images of viable cells taken by the optical microscope depict a minority of spherical cells that represent the dead cells, while most of the oval cells represent the viable cells. On the other hand, the concentration of viable cells is shown in Figure 8 after culturing the scaffold for 72 h. The concentration of Sm_2_O_3_/MgO/GO@CA started from 7500 µg/ mL. In addition, the IC_50_ is obtained at 6 mg/mL, which has the ability to degenerate the viable cells with a concentration of 50%. After the addition of nanoparticles to CA, the concentration was determined at 20 mg/mL, and the cell growth reached around 106%. As a result, there is a reversible relation between cell growth and component concentration. The additional components of nanoparticles seem to exhibit significant effects in improving the safety of the scaffolds towards the normal cells [35,36,37].

## 4. Conclusions

Nanoparticles with different ratios, including samarium oxide (Sm_2_O_3_) and magnesium oxide (MgO), were added to CA films and were fabricated by casting technique. Structural investigation via the X-ray diffraction technique (XRD) detected the cubic symmetry of MgO and Sm_2_O_3_. Further, the amorphous nature of CA was also detected. The result of the scanning electron microscope (SEM) represents the surface topography of CA with metal oxides, which have medium roughness and porosity with diameters in the range of 1.5–5 µm and 0.5–5 µm. The contact angle of the cast film indicated a noticeable change in the hydrophilic behavior with values from 27.9° to 29.4°. The cell viability test for Sm_2_O_3_/MgO/GO@CA films showed that the lowering in the concentration started from 7500 to 20 µg/mL, and the growth reached 106% after culturing the films for three days. The relatively high values of cell viability with good control of hydrophilicity might recommend these compositions for potential intensive applications, including dressing materials. The significant development of the physicochemical properties of CA scaffolds via the additional modifications indicates that Sm_2_O_3_ and MgO might provide a strategy to support the biological biocompatibility and, thus, the bioactivity of these scaffolds to be more appropriate for smart bandages and other potential clinical applications.

## Figures and Tables

**Table 1:** FT-IR bands (in cm^−1^) of CA films with different additives of nanocomposites.

CA	Sm_2_O_3_@CA	MgO@CA	Sm_2_O_3_/MgO@CA	Sm_2_O_3_/MgO/GO@CA	Assignment	Ref.
…	…	…	464	…	Mg–O–Mg	[32]
…	…	476	…	…	Mg–O–Mg	[32]
…	…	558	…	…	Mg–O–Mg	[32]
		658	…	656	MgO vibrations	[19]
902	903	902	…	901	C–OH stretch.	[27]
1025	1032	1028	…	1027	C–O	[8]
…	…	…	1054	…	C–O	[8]
1159	1160	1159	…	…	–CH group	[28]
1212	1219	1216	1216	1219	(C-O-C)	[8]
1369	1369	1368	…	1371	(C-CH3)	[8]
1426	1429	1426	…	1425	–CH	[28]
…	…	…	…	1558	C=C bonds	[36]
1662	1662	1661	…	1658	C=O	[27]
1742	1745	1738	1746	1742	carbonyl	[29]
…	…	…	…	2926	C–H stretching	[34]
2943	2941	…	…	…	– CH_2_ stretching	[30]
…	…	…	2972	…	C–H stretching	[34]
…	…	…	3393	…	(–OH) group	[33]
…	…	3439	…	…	(–OH) group	[33]
…	3454	…	…	…	(–OH) groups	[13]
3742	…	…	3739	…	(-OH) stretching vibration	[31]
…	…	…	3836	…	O–H	[35]

**Table 2:** EDX examination for compositions Sm_2_O_3_/MgO/GO@CA.

Elements	Weight %	Atomic %
C K	43.17	51.14
N K	3.59	3.65
O K	48.84	43.45
MgK	2.74	1.6
SmL	1.66	0.16

## Data Availability

The raw/processed data generated in this work are available upon request from the corresponding author.
